# microRNA-497 slows esophageal cancer development and reverses chemotherapy resistance through its target QKI

**DOI:** 10.18632/aging.204713

**Published:** 2023-05-11

**Authors:** Yun-Xia Xie, Zhi-Hao Zhou, Shu-Wen Liu, Ye Zhang, Wen-Jing Liu, Rui-Ke Zhang, Ming-Liang He, Jian-Ge Qiu, Lin Wang, Bing-Hua Jiang

**Affiliations:** 1 Academy of Medical Science, Zhengzhou University, Zhengzhou 450000, China; 2 BGI College and Henan Institute of Medical and Pharmaceutical Sciences, Zhengzhou University, Zhengzhou 450000, China; 3 The Affiliated Cancer Hospital of Zhengzhou University and Henan Cancer Hospital, Zhengzhou University, Zhengzhou 450000, China; 4 Department of Biomedical Sciences, City University of Hong Kong, Hong Kong 999077, Hong Kong

**Keywords:** miRNA-497, QKI, esophageal cancer, chemotherapy resistance, tumor growth

## Abstract

Esophageal cancer (EC) is considered one of the most lethal cancers in human beings, and multiple miRNAs have been investigated to be involved in EC development by targeting their target genes. However, the function and related mechanism of miRNA-497 on EC tumorigenesis remain uncertain. This study first demonstrated that the expression levels of miR-497 in esophageal cancer specimens and cells were down-regulated. Forced expression of miR-497 inhibited cell proliferation, tube formation and migration in EC cells. To further investigate the potential molecular mechanism of miR-497 suppression in regulating EC, we found that miR-497 directly binds to the 3'-untranslational region of QKI, miR-497 overexpression suppressed QKI expression. We further found that overexpression of miR-497 enhanced the effect of chemotherapy in EC cell lines, and prevented the tumor growth of EC *in vivo*. Our findings indicated that miR-497 suppression increased QKI expression and therapeutic resistance of esophageal cancer, which is likely to be a biomarker of EC progression and potential therapeutic target.

## INTRODUCTION

Esophageal cancer is considered one of the most common malignancies in human digestive system [1]. Despite advances in the esophageal cancer management and therapy in patients, the 5-year overall survival rates (~10%) and postesophagectomy survival rates (~15-40%) remain quite low [2]. Esophageal cancer is often first diagnosed at advanced clinical stages, due to mostly lack of early clinical obvious symptoms in patients [3, 4]. Identification of susceptible genes or biomarkers will be valuable to predict the therapy response of EC patients for promoting the overall survival rates of patients. Therefore, investigating new molecular mechanisms of EC development is urgently needed.

MicroRNAs (miRNAs) are noncoding RNAs in cells with 18-25 nucleotide that are highly conserved in humans and are endogenous biological regulators of multiple gene expression [5–7]. MiRNAs typically interact with the 3′-UTRs (3′-untranslated regions) of target genes’ mRNAs (messenger RNAs), triggering miRNA cleavage and then degrading the indicated mRNA or inhibiting the translation process. MiRNAs are reported to involved in several cancer development processes, including tumor initiation, metastasis, drug resistance and tumor recurrence [8–10]. In addition, miRNAs are represented as potential biomarkers for early diagnosis or/and therapeutic targets for treatment of cancer. MiR-497 dysregulation has been associated with several cancers, including a study showing that miR-497 blocked the activation of AKT2 gene and increased chemosensitivity of lung cancer cells to cisplatin treatment [11]. MiR-497 was found to be overexpressed in glioma and forced expression of miR-497 promoted chemotherapy resistance of glioma cells to Temozolomide treatment [12]. However, the role and mechanism of miR-497 in EC are unclear yet. Here, we first investigated the role of miR-497 in EC and its underlying mechanism for regulating EC tumorigenesis.

QKI (quaking), member of the STAR family, contains the KH domain and works as an RNA-binding protein [13]. Recent studies suggested that the loss or overexpression of QKI was related to the process of various human disorders or/and diseases including muscle-differentiation, diabetic heart disease, and cancers [14–16]. One study showed that QKI regulated the TGF-β-induced EMT process of human epithelial cells [17]. A previous study reported that QKI was a target of miR-574 and potentially involved in cervical cancer progression and cell metastasis [18]. The role of miR-497-QKI pathway in esophageal cancer cells remains unclear.

Thus, in this study, we address the following questions: (a) whether miR-497 expression is associated with esophageal cancer incidence; (b) what the underlying role of miR-497 in tumor progression is; (c) whether miR-497 induces chemosensitivity to drug treatment in esophageal cancer; (d) what direct target of miR-497 is for regulating esophageal cancer; and (e) whether expression levels and correlation between miR-497/QKI may have the prognostic value in esophageal cancer, or be used for developing new prognostic markers and treatment option in the future.

## MATERIALS AND METHODS

### Esophageal cancer specimens

Paired human esophageal cancer (EC) tissues and adjacent normal specimens were obtained from The Biobank of the Linzhou Cancer Hospital, Henan, China. The specimens' collection of Biobank was approved by Ethics Committee of Zhengzhou University. The Biobank has been collecting cancer tissues for many years from the clinical patients who underwent surgery. The human specimens were immediately frozen in liquid nitrogen (LN) and stored in the Biobank. For the experiments in this study, cancer tissues were obtained and used without patient information.

### Cell culture and reagents

Human esophageal cancer cells Eca109 and Kyse30 were grown in RPMI 1640 medium (Invitrogen, Waltham, MA, USA), and HEK-293T cells (human embryonic kidney 293T) were cultured in DMEM medium (Invitrogen, Waltham, MA, USA) supplemented with 10% FBS (fetal bovine serum), 100 U/ml penicillin plus 100 ng/ml streptomycin (Gibco Life Technology, New York, NY, USA). All human cell lines were cultured at 37° C with 5% carbon dioxide.

### RNA isolation and quantitative PCR analysis

Total RNA in human cells or tissues was isolated with the Trizol reagent following instructions of manufacturer (Thermo Fisher Scientific, Waltham, MA, USA). For each human sample, RNA (0.5 μg) was reverse transcribed by the RT Kit (Vazyme, Nanjing, China). SYBR Green PCR Mix (Vazyme, Nanjing, China) was used for quantitative PCR analysis. The U6/miR-497 RT and PCR primers were purchased from RiboBio company (Guangzhou, China). All treatments were performed in triplicate and 2^-(∆∆Ct) method was used to analyze the relative expression of indicated genes in each sample.

### Western blotting

After washing cells with 1× PBS buffer in a 6cm cell culture dish, cells were fully lysed with RIPA lysis buffer and then total proteins were collected. Equal amounts of total protein in indicated samples were separated by SDS-PAGE gels and then transferred onto NC membrane (nitrocellulose membrane). Indicated membranes were incubated with the indicated target antibodies. Then immunoreactivity of indicated band was detected by enhanced chemiluminescence (ECL) (Thermo Fisher Scientific, Waltham, MA, USA) and the images were analyzed using Image J software. Antibodies were commercially obtained in this study as below: the anti-QKI antibody was purchased from Cell Signaling Technology (Danvers, MA, USA); the anti-GAPDH antibody was obtained from Bioworld Technology (Atlanta, GA, USA).

### Dual-luciferase reporter assay

Dual-luciferase reporter assay was purchased from Promega (Madison, WI, USA). The luciferase reporters containing the 3′-UTR regions of QKI gene with the wild-type (WT) or mutant (MT) binding sites of miR-497 were constructed separately. The reconstructed luciferase reporter plasmids have been sequenced and validated. Then luciferase assay kit was used to measure luciferase activity 24 hours after transfection of reporter plasmids into cells. The activity of the Renilla luciferase in cells was used as the control. The experiments were carried out in three independent replicates.

### Clonogenicity assay

The cells were collected and the number of cells was counted. 500 cells were then seeded into 12-well plates per well and evenly distributed. After incubation for 10-14 days in cell culture medium supplemented with 10%FBS, cells formed visible colonies, and these colonies were stained with 0.005% crystal violet (containing 20% methanol) and were counted using Image J Software.

### Transwell and tube formation assay

Transwell chambers (8 μm) were purchased with Corning (New York, NY, USA). Cells (1×10^5^) were carefully collected and washed twice with 1×PBS buffer. Then cells were resuspended with 200 μL serum-free culture medium and added to the upper chamber. 500μL medium with 10% FBS was placed in lower chamber. After incubation at 37° C incubator for 24 hours, the cells were stained and fixed with methanol and crystal violet for 30 minutes. The amounts of migrating cells were analyzed with a microscope. Crystal violet stained cells were eluted using 33% acetic acid, and the absorbance values of cells at OD 570 nm were measured. Tube formation assay was performed as we previously described [19].

### Tumor xenograft model

BALB/c female nude mice were purchased from GemPharmatech company (Nanjing, China), and bred in SPF (special pathogen-free) condition. 5×10^6^ cells were collected and injected into both flanks of the nude mice. Ago-miR-497 is a specially chemically modified miR-497, which can be directly injected into animals to have similar effect as endogenous miR-497 (RiboBio company, Guangzhou, China). Ago-miR-NC is the negative control. After the tumors were clearly detected, Ago-miR-497 and Ago-miR-NC were intratumorally injected into the mice tumors every two days and indicated tumor volumes were measured. Tumor volumes (mm^3^) were calculated as follows: V = 1/2×Length×Width^2^. Mice were euthanized and tumor xenografts were harvested. Parts of the specimens were immediately fixed in 10% formalin and further embedded in paraffin, the other parts of specimens were analyzed by immunoblotting. Animal experiments were conducted following along Guidelines of National Institutes of Health.

### Immunohistochemical staining

Tumor tissue sections (5 μM) were made and used for immunohistochemical analysis. After paraffin removal, antigen repair, and removal of peroxidase from the sections, sections were incubated with indicated primary antibodies. Then slides were stained using DAB solution (diaminobenzedine, MXB Biotechnologies, Fuzhou, China), then counterstained with haematoxylin. Slices were photographed with Image Analyse system of Leica Microscope. Four low-power views (200×) were randomly selected from each slice.

### Pathway enrichment analysis

Data of 83 esophageal squamous cell carcinoma samples was download from The Cancer Genome Atlas (TCGA). The total samples were divided into 2 groups (QKI with lower expression group and QKI with higher expression group) based on the one-quarter and three-quarters values (25% and 75%) of QKI in all samples. Then, the differentially expressed genes between 2 groups were analyzed by DESeq2 package in R software (version 3.6.1). The threshold of differentially expressed genes is p value < 0.05 and the absolute value of log2 fold change>1 between the two groups. KEGG and GO (MF) pathway enrichment analysis was performed on all differentially expressed genes. The threshold of pathway enrichment is adjusted p value (Benjamini-Hochberg) < 0.05, q value <0.2 and others are default parameters.

### Gene set enrichment analysis

Gene Set Enrichment Analysis (GSEA) program (4.0.2) was used to analyze the differences in biological processes between QKI low expression group and QKI high expression group using differentially expressed potential genes between the two groups. The enrichment pathways from the molecular signatures database (MSigDB) include HP_ABNORMAL_CELL_PROLIFERATION and HALLMARK_TGF_BETA_SIGNALING. The threshold of pathway enrichment was p value < 0.05.

### Statistical analysis

Data analysis in this study were presented as mean ± SD. GSE114110 dataset was re-analyzed by limma package in R software (version 3.6.1). P values were measured by one-way ANOVA method or Student's *t*-test method for unpaired samples by GraphPad Prism software (La Jolla, CA, USA). The results were considered to be significantly different at P<0.05.

### Data availability statement

All datasets and the public databases GEO and TCGA will be provided upon the request included within the article. (https://www.ncbi.nlm.nih.gov/geo/query/acc.cgi?acc=GSE114110; https://www.ncbi.nlm.nih.gov/geo/query/acc.cgi?acc=GSE43732).

## RESULTS

### miR-497 expression is down-regulated in the clinical samples of EC patients

**Figure 1:**
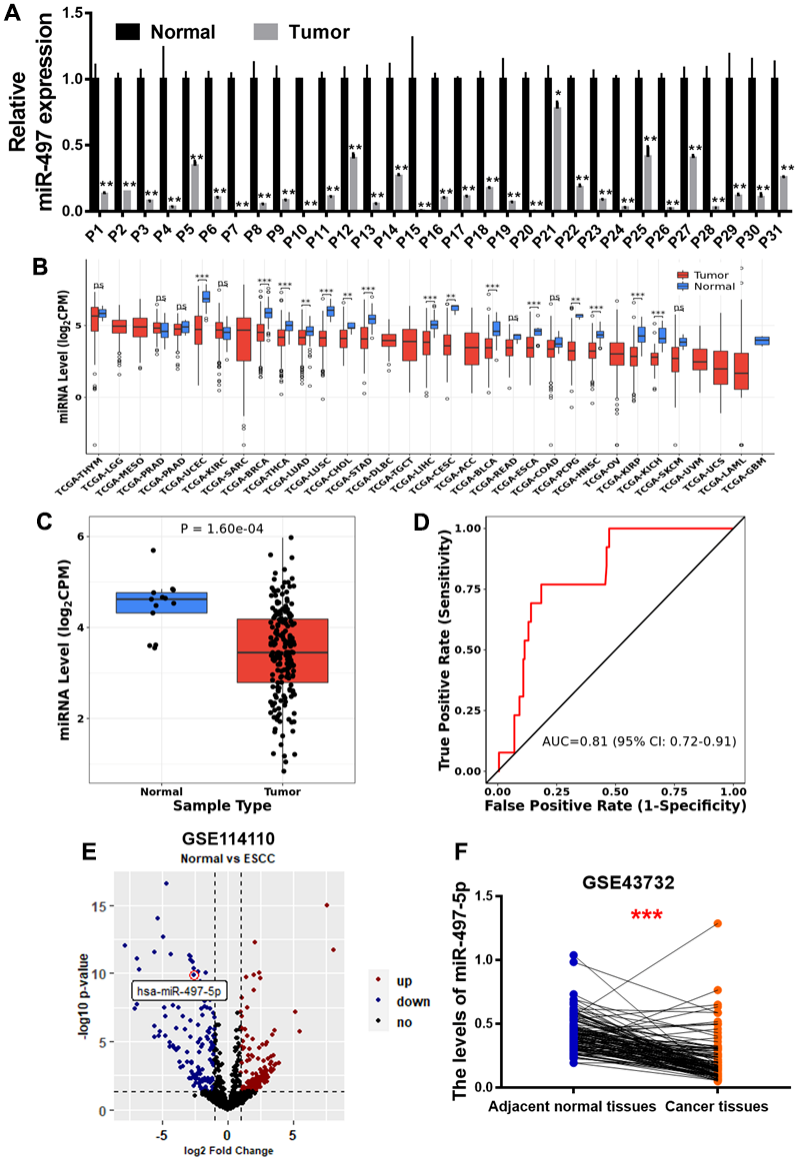
**miR-497 expression is down-regulated in the tumor tissues of EC patients.** (**A**) Compared to adjacent normal tissues, the expression levels of miR-497 in EC tissues were significantly lower. (**B**, **C**) The result of CancerMIRNome (http://bioinfo.jialab-ucr.org/CancerMIRNome/) database showed that miR-497 levels were much lower in several types of cancer tissues, especially in esophageal cancer. (**D**) ROC curve showed that miR-497 may be used as a biomarker in EC prognosis. (**E**) Volcano plot of GSE114110 displayed differentially expressed microRNAs in normal and EC tissues. (**F**) Analysis of GSE43732 showed that miR-497 expression levels were significantly lower in EC samples. Data were representative of 3 independent experiments. * indicated significant difference at p<0.05, ** indicated significant difference at p<0.01, *** indicated significant difference at p <0.001; ns indicated p >0.05.

To explore miR-497 expression levels in the EC tissues, we first analyzed 31 pairs of tumor and adjacent normal specimens from the EC patients, detected miR-497 levels using q-RT-PCR assay, and found that miR-497 expression levels were much lower compared to those in the adjacent normal specimens (Figure 1A). Then, we analyzed miR-497 expression levels in different types of cancer in TCGA database, and found that miR-497 expression levels were down-regulated in multiple types of cancer (Figure 1B), especially in esophageal cancer (Figure 1C). The ROC (receiver operating characteristic) curve also showed that miR-497 expression levels may be showed as a potential biomarker in esophageal cancer (Figure 1D). Similarly, miR-497 expression levels in EC tissues were significantly down-regulated compared to those in normal tissues using two different sets of Gene Expression Omnibus (GEO) database (GSE114110, GSE43732) (Figure 1E, 1F). Furthermore, we have analyzed the TCGA and GEO database of EC patients, and suggested that there was no obvious difference of miR-497 expression levels in the subjects among different gender, age, or weight (Supplementary Figure 1A–1C) using TCGA data. Similar results were obtained by analyzing GSE114110 and GSE43732 datasets (Supplementary Figure 1D, 1E). All these results both suggest that miR-497 may act as a tumor suppressor gene and a potential new biomarker in diagnosis of esophageal cancer.

### miR-497 overexpression inhibits cell proliferation, migration, tube formation and colony formation

**Figure 2:**
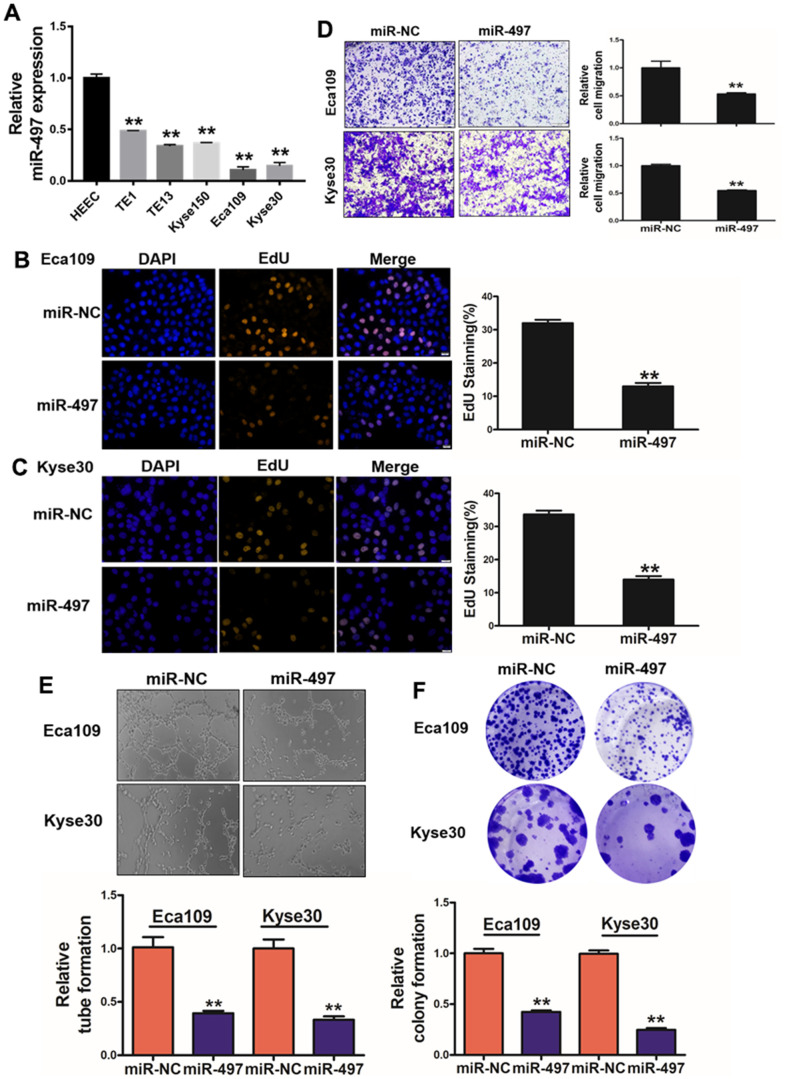
**miR-497 overexpression inhibits cell proliferation, migration, tube formation and colony formation.** (**A**) Compared to normal esophageal HEEC cell, the expression levels of miR-497 in EC cell lines were significantly lower. (**B**, **C**) Overexpression of miR-497 greatly reduced percentages of EdU staining signals in Eca109 and Kyse30 cells. (**D**) Overexpression of miR-497 inhibited cell migration activities. (**E**) The overexpression of miR-497 attenuated tube formation activities. (**F**) Forced expression of miR-497 attenuated the colony formation activities in cancer cells. All data were representative of 3 independent experiments. ** indicated significant difference at p<0.01.

We detected miR-497 expression levels in human esophageal epithelial cell (HEEC) and five esophageal cancer cell lines (TE1, TE13, Kyse150, Eca109 and Kyse30), and the result conveyed that expression levels of miR-497 were significantly lower in esophageal cancer cells than those in HEEC cells (Figure 2A). To test functional role of miR-497 on the cell proliferation and migration activity in esophageal cancer cell lines, we showed overexpression of miR-497 significantly inhibited cell proliferation and migration activities (Supplementary Figure 2 and Figure 2B–2D). As we expected, overexpression of miR-497 also inhibited tube formation activity using HUVECs (human umbilical vein endothelial cells), and decreased the colony formation ability (Figure 2E, 2F). Thus, our results demonstrate that miR-497 overexpression inhibits multiple cell functions of esophageal cancer.

### miR-497 suppression is mediated by EZH2 and histone acetylation

**Figure 3:**
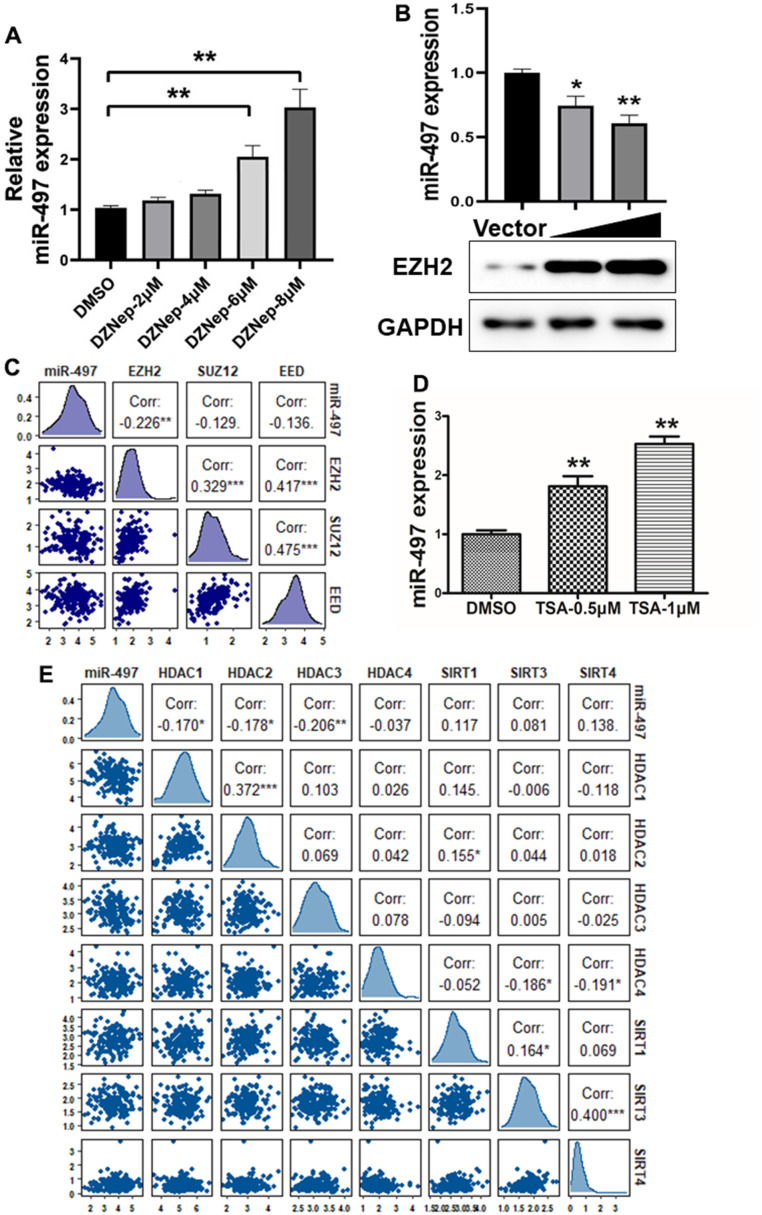
**miR-497 suppression is mediated by EZH2 and histone acetylation.** (**A**) Cells were treated with EZH2 inhibitor DZNep, and DZNep treatment greatly increased miR-497 expression levels in the cells in a dose-dependent manner. (**B**) Compared to vector control, overexpression of EZH2 significantly reduced miR-497 expression. (**C**) A negative correlation was showed between EZH2 and miR-497 levels in esophageal cancer tissues from the data obtained from TCGA database. (**D**) The histone deacetylases inhibitor TSA treatment in Eca109 induced miR-497 expression levels. (**E**) The esophageal cancer data from TCGA was used to analyze correlation between expression levels of miR-497 and histone deacetylases: HDAC1, HDAC2 and HDAC3, which is inversely correlated. Spearman’s correlation analysis was carried out in (**C**, **E**). All data were representative of 3 independent experiments. * indicated significant difference at p<0.05, ** indicated significant difference at p<0.01.

Histone modifications as crucial epigenetic alterations associated with chromatin remodeling were identified to regulate miRNA expression levels in EC [20, 21]. EZH2 (Enhancer of Zeste Homolog 2), a core subunit of PRC2 (polycomb repressive complex 2), has been reported as a key epigenetic regulator of histone methylation involved in cancer progression [22]. We found that EZH2 levels are significantly upregulated in esophageal cancer tissues compared to normal tissues (Supplementary Figure 3A). To identify mechanism of miR-497 suppression in EC, we found that the cells treated with the EZH2 inhibitor, DZNep, increased miR-497 levels, whereas EC cells with forced expression of EZH2 greatly reduced miR-497 expression levels (Figure 3A, 3B). We then analyzed TCGA database to evaluate the association between the expression levels of miR-497 and PRC2 complex, which includes EZH2, EED and SUZ12, and the results showed that there was an inversely strong correlation between levels of miR-497 and EZH2 compared to SUZ12 and EED levels, indicating that EZH2 was the limited regulator of miR-497 expression in esophageal cancer (Figure 3C).

To further study whether histone acetylation modification may be engaged in miR-497 suppression, we showed that miR-497 expression was significantly induced by the treatment of HDAC (histone deacetylase) inhibitor trichostatin A (TSA) in the cells (Figure 3D), suggesting that histone acetylation modification may be involved in regulating miR-497 expression. Histone deacetylases are important in regulating the balance of histone acetylation modification in cancer progression. We then analyzed TCGA database to investigate the relationship between miR-497 and histone deacetylases, including HDAC and SIRT families. The expression levels of HDAC1, HDAC2 and HDAC3 were significantly up-regulated in esophageal cancer specimens compared to normal specimens (Supplementary Figure 3B–3D). We found that HDAC1, HDAC2 and HDAC3 expression levels were obvious negatively correlated with miR-497 expression levels in TCGA database, but not with levels of HDAC4 and SIRT family members (Figure 3E). Our study showed that both tumor-specific histone methylation and higher expression levels of histone deacetylases HDAC1, HDAC2 and HDAC3 are involved in miR-497 downregulation in esophageal cancer cells, suggesting the epigenetic complexity of miRNA deregulation.

### miR-497 overexpression increases the effect of 5-FU, CDDP and paclitaxel treatment in EC cells

**Figure 4:**
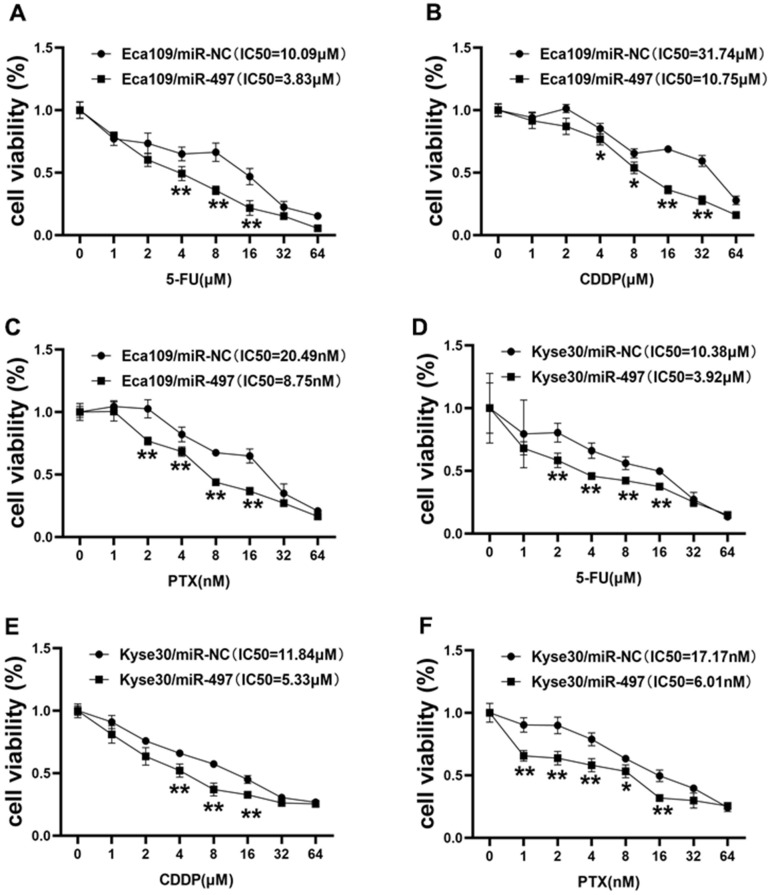
**miR-497 overexpression increases the effect of 5-FU, CDDP and paclitaxel treatment in EC Cells.** (**A**–**C**) Eca109 cells were treated with different concentrations of 5-FU, CDDP or paclitaxel as indicated, the cell viability was analyzed using CCK8 assay. Overexpression of miR-497 significantly increased chemo-sensitivity. (**D**–**F**) Forced expression of miR-497 in Kyse30 rendered cells more sensitive to 5-FU, CDDP or paclitaxel treatment. Data were representative of 3 independent experiments. * indicated significant difference at p<0.05, ** indicated significant difference at p<0.01.

During clinical treatment of esophageal cancer, 5-fluorouracil (5-FU), cisplatin (CDDP), and paclitaxel (PTX) are traditionally used in patients. However, chemoresistance to these agents represents the main obstacle in therapeutic treatment of esophageal cancer. In this study, we firstly measured the cell viability to reflect cell sensitivity to drug treatment using the CCK-8 kit, and the cell viability results conveyed that Eca109 cells transfected with miR-497 was inhibited after 5-FU, CDDP or PTX treatment, and the IC50 values of miR-497 group were significantly decreased (Figure 4A–4C). Then, we further analyzed the drug sensitivity in another different esophageal cancer cell line Kyse30, and our results showed that forced expression of miR-497 increased the cell sensitivity to 5-FU, CDDP or PTX treatment in Kyse30 cells, and overexpression of miR-497 in the cells showed decreased IC50 values (Figure 4D–4F). These results show that miR-497 expression increased drug sensitivity in esophageal cancer cells.

### QKI is a direct target of miR-497, and QKI expression levels were correlated with tumor progression in EC samples

**Figure 5:**
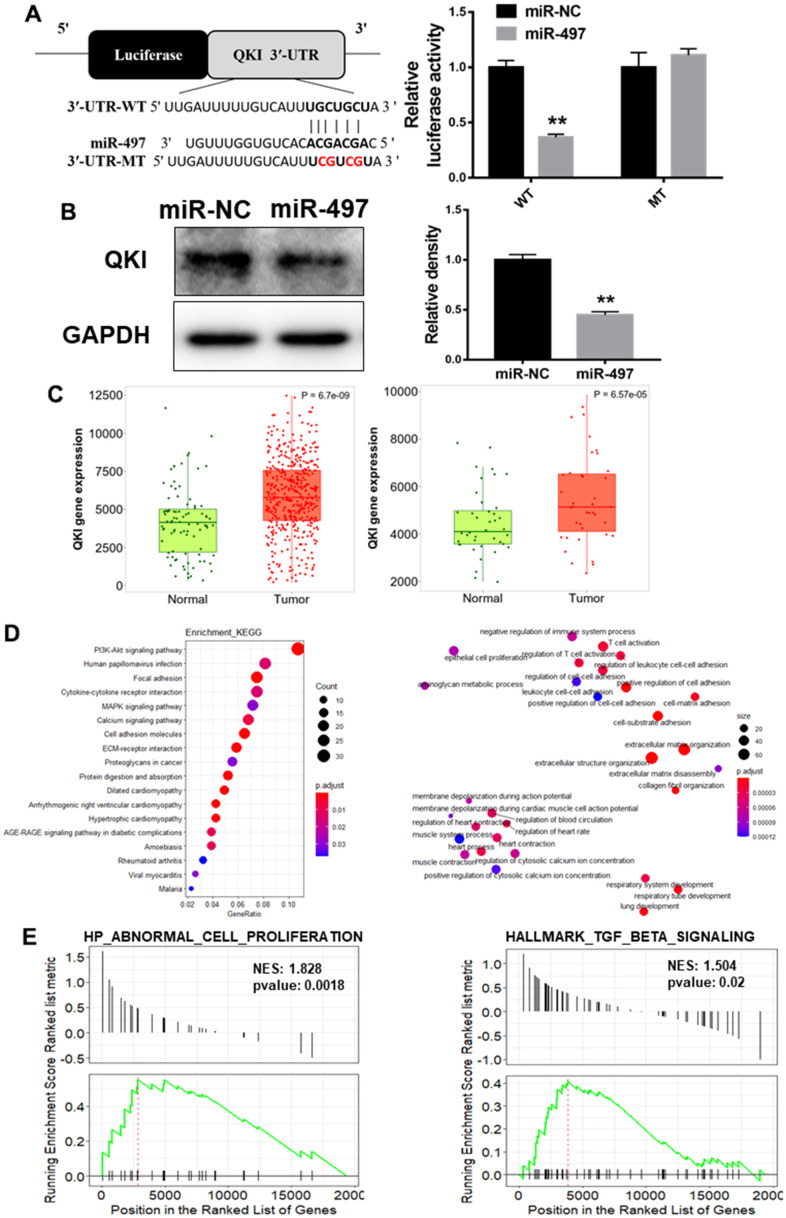
**QKI is a direct target of miR-497, and QKI expression levels were correlated with tumor progression in EC samples.** (**A**) Overexpression of miR-497 inhibited the luciferase activity of QKI 3’ UTR reporter. (**B**) Overexpression of miR-497 greatly inhibited protein expression levels of QKI. (**C**) The expression levels of QKI were significantly upregulated in non-paired and paired EC tissues compared to the normal ones. (**D**) KEGG and GO (MF) analysis of esophageal cancer tissues showed that QKI is associated with cell proliferation and cell adhesion signal pathways. (**E**) GSEA analysis of esophageal cancer tissues conveyed that QKI expression levels were positively correlated with cell proliferation and metastasis in EC tissues. Data were representative of 3 independent experiments. ** indicated significant difference at p<0.01.

To investigate the mechanism of miR-497 downregulation in EC progression, QKI was candidate target of miR-497 based on the pairing of the seed binding sequence of miR-497 (Figure 5A). To determine whether miR-497 regulated QKI expression through the binding to 3′-UTR, we constructed the wild (WT) or mutant (MT) luciferase reporter plasmids with the potential binding site of QKI 3′-UTR and performed dual luciferase reporter assay. As result shown in Figure 5A, miR-497 overexpression decreased the luciferase activities of WT reporter plasmids, but not the MT plasmids. Further study showed that QKI expression levels were greatly decreased in the cells with miR-497 overexpression (Figure 5B). Then TNM plot database was used to analyze QKI expression in unpaired and paired esophageal cancer samples, and QKI levels were showed to be upregulated in both unpaired and paired samples (Figure 5C). GO (Gene Ontology) analysis and KEGG (Kyoto encyclopedia of genes and genomes) functional enrichment analysis were applied to predict potential biological processes and downstream signaling pathways mediated by QKI (Figure 5D). These results of pathway enrichment analysis revealed that QKI expression was mainly associated with several biological process related to cancer progression, including PI3K/AKT signaling activation and cell adhesion. Then, Gene Set Enrichment Analysis (GSEA) based on QKI differential expression data from TCGA was performed. As shown in Figure 5E, the QKI expression levels were positively correlated with the proliferation and metastasis-associated gene signatures. Thus, the results demonstrate that QKI is a direct target of miR-497, playing as the oncogenic role in the progression of EC.

### miR-497 overexpression inhibits tumor growth and QKI expression *in vivo*

**Figure 6:**
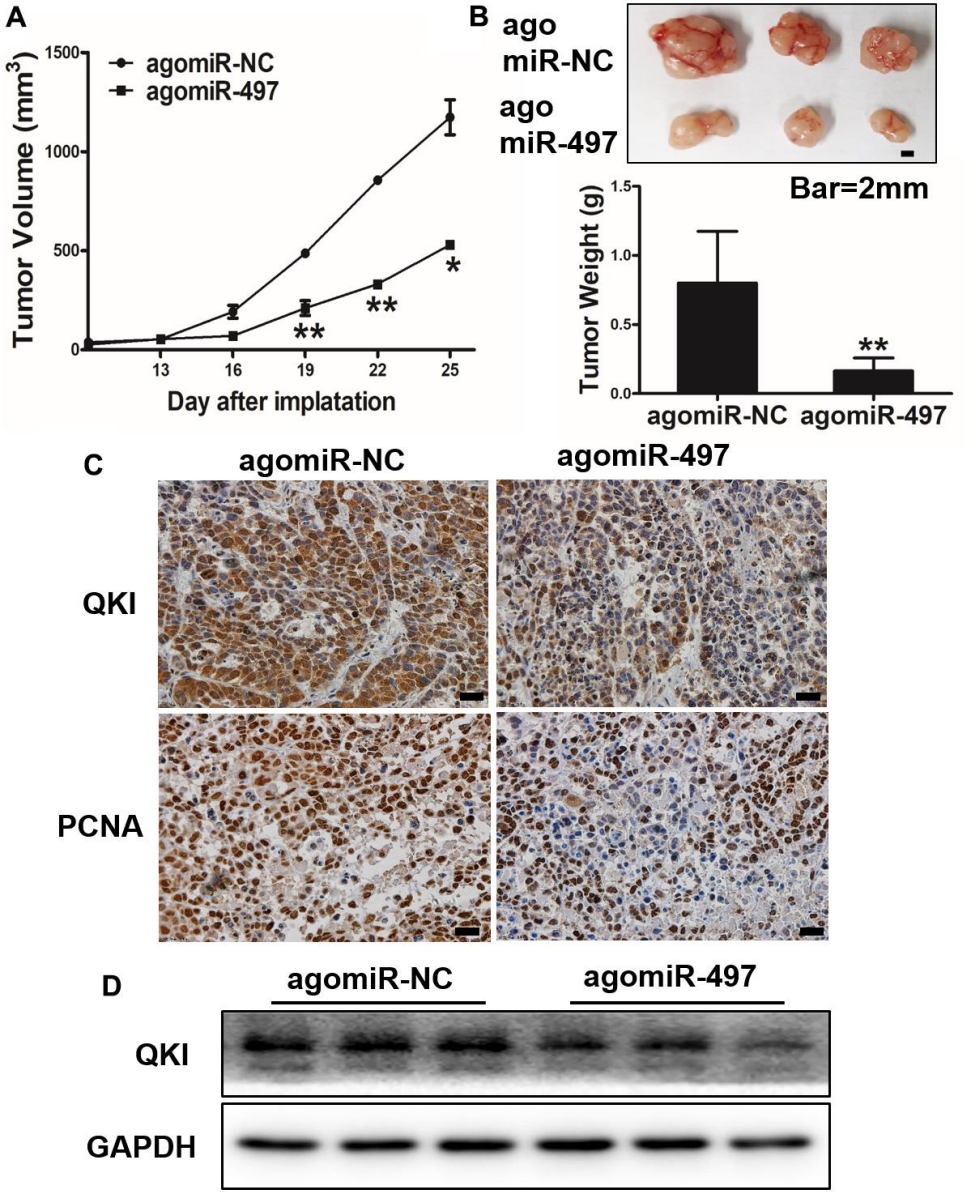
**miR-497 overexpression inhibits tumor growth and QKI expression *in vivo*.** (**A**, **B**) Overexpression of miR-497 inhibited tumor growth. The photograph showed the representative tumors from each group. Scale bar, 2 mm. Tumor weights from miR-497 overexpression group were significantly decreased compared to control group. Scale bar, 20 μm. (**C**) Immunohistochemistry (IHC) assay indicated that overexpression of miR-497 inhibited the expression levels of QKI and PCNA. (**D**) The expression levels of QKI from different tumor tissues as indicated were analyzed by immunoblotting. Data were representative of 3 independent experiments. * indicated significant difference at p<0.05, ** indicated significant difference at p<0.01.

Next, we investigated whether miR-497 and its target QKI may affect tumorigenesis and progression *in vivo*. Esophageal cells were inoculated subcutaneously into the both flanks of female nude mice. Then, Ago-miR-NC or Ago-miR-497 was injected into indicated tumors every 2 days. Tumor volumes were measured on Days 13, 16, 19, 22, and 25 to evaluate the growth curve of tumors. As results shown in Figure 6A, 6B, Ago-miR-497 (miR-497) overexpression displayed a significant reduction of tumor growth *in vivo*. Histologic analysis indicated that tumor samples derived from Ago-miR-497 treatment had a significantly decreased QKI expression than Ago-miR-NC tumors, similarly the proliferation marker PCNA was also inhibited (Figure 6C). Compared to Ago-miR-NC group, Ago-miR-497 significantly inhibited QKI levels by Western blotting (Figure 6D). Together, these results suggest that miR-497 inhibits EC tumor growth via targeting QKI *in vivo*.

## DISCUSSION

A large number of recent researches investigated that miRNAs have an important role in various physiological processes, including cell proliferation, differentiation as well as apoptosis [7–9, 23]. MiRNAs have considered to function as tumor suppressors or oncogenes based on their targets and play vital role in the tumor initiation and development. Recent report showed the role of miR-502 in regulating the cell proliferation through promoting AKT phosphorylated levels in esophageal cancer cell [24]. Although a variety of miRNAs have been found to be engaged in the development of esophageal cancer, the role of miR-497 in esophageal cancer remains not fully understood. This research aims to analyze the role and molecular mechanism of miR-497 in regulating esophageal cancer.

Accumulating studies from our group and other researchers demonstrated that epigenetic regulation, including DNA methylation and histones modification, promotes cancer development and progression [25–27]. In addition, recent study showed that miR-493/Wnt5A/c-JUN regulatory axis as a novel feedback loop that affected the esophageal cancer development, and miR-493 regulated Wnt5A, inhibited c-JUN activity, and enhanced p21 expression, whereas c-JUN bonded to the upstream promoter region of miR-493 to suppressing miR-493 expression, then forming a vital negative feedback loop which provides potential targets for therapy of esophageal cancer [28]. In this study, EZH2 promoted miR-497 silencing, as well as HDAC inhibitor induced miR-497 expression levels, which firstly identified that histone modification via HDACs and EZH2 are main regulators in miR-497 downregulation in esophageal cancer. Thus, epigenetic regulation study of miR-497 will improve understanding of the molecular basis of EC, and will provide valuable strategies to optimize EC treatment in the future.

Drug resistance represents the major obstacle in therapy of esophageal cancer. Increasing evidence shows that drug resistance is closely associated with miRNA dysregulation. Overexpressed miR-218 in human lung cancer cells increased chemosensitivity to cisplatin therapy through down-regulating ZEB2 and Slug expression [29]. In this study, we identified that miR-497 overexpression greatly increased the effect of 5-FU, CDDP and paclitaxel treatment to inhibit the proliferation of Eca109 and Kyse30 cells, suggesting that different miRNAs are important in cancer cell resistance to drug treatments. Therefore, it is crucial to identify specific targets for clinical diagnosis and treatment evaluation of esophageal cancer in the future. The finding that the molecular mechanism of miR-497/QKI axis in inhibiting chemoresistance in esophageal cancer will promote further study of miR-497 and QKI as new biomarkers or therapeutic targets of esophageal cancer in the future.

## Supplementary Material

Supplementary Figures
