# Supplementary material for: microRNA-497 slows esophageal cancer development and reverses chemotherapy resistance through its target QKI
Source: Aging (Albany NY). 2023 May 11;15(9):3791–806. doi: 10.18632/aging.204713 (PMC10449293; doi:10.18632/aging.204713)
Supplement: Supplementary Figures [file aging-15-204713-s001.pdf]

SUPPLEMENTARY FIGURES

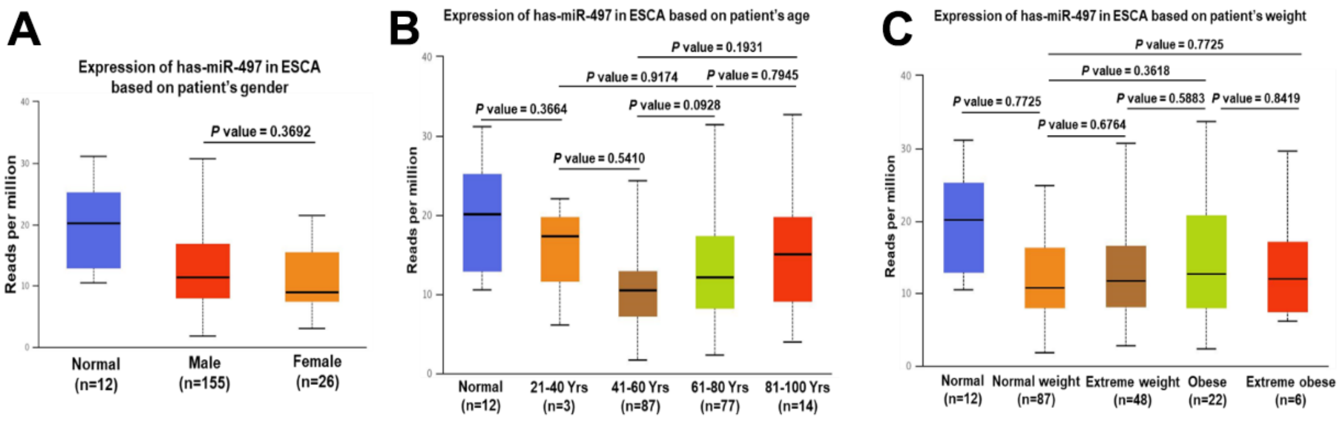

D

GSE114110			
	Normal	ESCC	p value
Samples	10	30	
Age(years)	60.7(average)	56.3(average)	0.06369 (Student's t-test)
Gender			
Male	7	27	0.3065(Chi-square test)
Female	3	3	

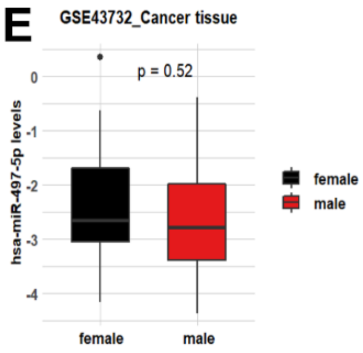

Supplementary Figure 1. The miR-497 expression levels in human esophageal cancer tissues in subjects with different gender, ages, or weight. (**A**–**C**) There was no significant difference of miR-497 expression levels in the subjects with different gender, ages, or weight using TCGA data. (**D**, **E**) The different levels of miR-497 levels were obtained by analyzing GSE114110 and GSE43732 datasets.

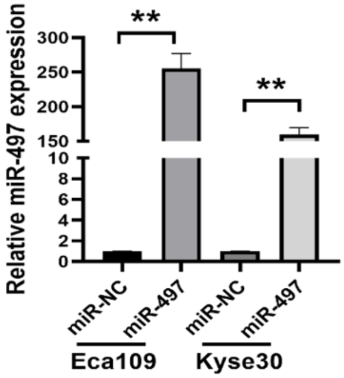

Supplementary Figure 2. Overexpression of miR-497 in Eca109 and Kyse30 cells. Q-PCR assay was used to analyze expression levels of miR-497 in esophageal cancer cells. Data were representative of 3 independent experiments. ** indicated significant difference of miR-497 levels compared to the control at p<0.01.

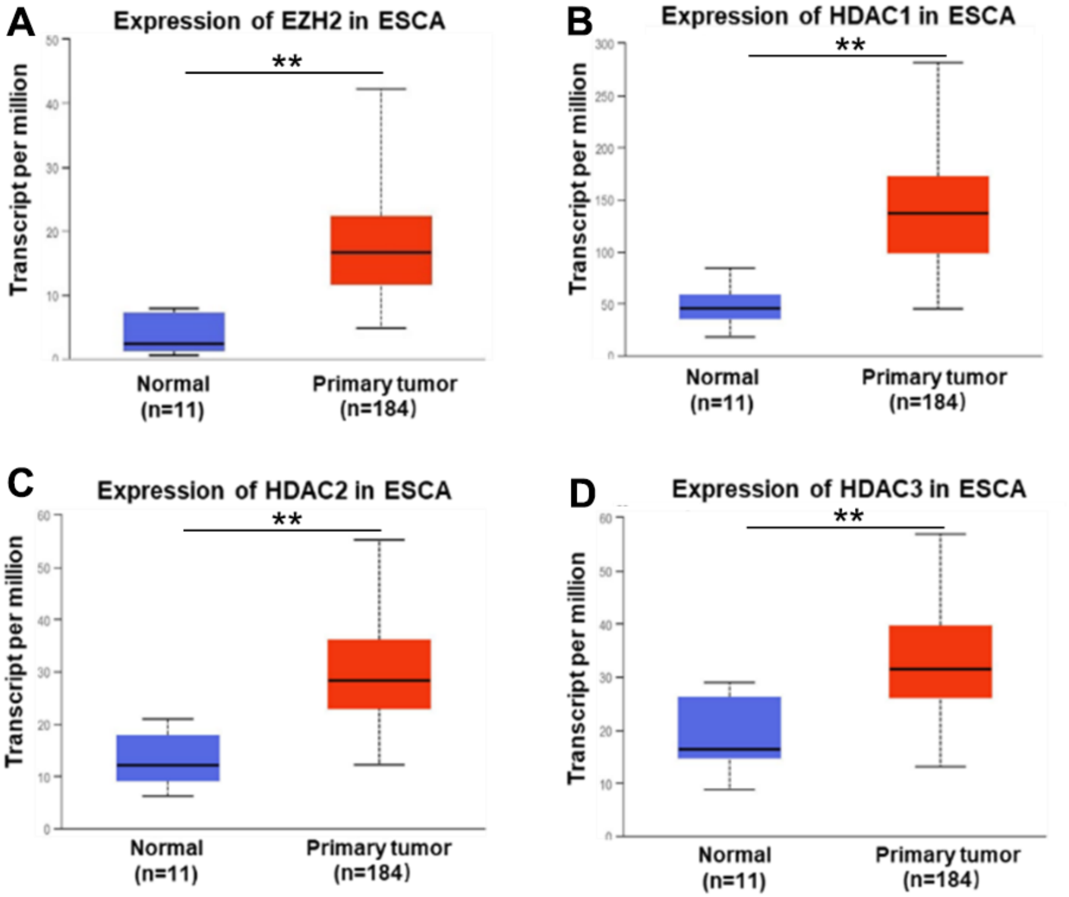

Supplemental Figure 3. The expression levels of HDACs and EZH2 in esophageal cancer tissues. (**A**–**D**) The expression levels of EZH2 and HDACs (HDAC1, HDAC2, HDAC3) were analyzed in esophageal cancer and normal tissues by using TCGA database. ** indicated significant difference of these molecule expression levels compared to the control at p<0.01.
